# Evaluating the impact of an oral care initiative on the risk of non-ventilator-associated hospital-acquired pneumonia using electronic clinical data and diagnostic coding surveillance criteria

**DOI:** 10.1017/ice.2025.54

**Published:** 2025-12

**Authors:** Barbara E. Jones, Alec B. Chapman, Jian Ying, McKenna R. Nevers, Shannon Munro, Michael Klompas, Amy L. Valderrama, Daniel O. Scharfstein

**Affiliations:** 1 Division of Pulmonary & Critical Care Medicine, University of Utah, Salt Lake City, UT, USA; 2 Veterans Affairs Salt Lake City Health Care System, Salt Lake City, UT, USA; 3 Department of Population Health Sciences, University of Utah, Salt Lake City, UT, USA; 4 Division of Epidemiology, University of Utah, Salt Lake City, UT, USA; 5 Research and Development, Department of Veterans Affairs Medical Center, Salem VA Medical Center, Salem, VA, USA; 6 Department of Population Medicine, Harvard Pilgrim Health Care Institute and Harvard Medical School, Boston, MA, USA; 7 Centers for Disease Control and Prevention, Atlanta, GA, USA; 8 Division of Biostatistics, Department of Population Health Sciences, University of Utah School of Medicine, Salt Lake City, UT, USA

## Abstract

**Objective:**

We assessed the impact of an oral care initiative on non-ventilator-associated hospital-acquired pneumonia (NV-HAP) risk using two different measurement strategies.

**Methods:**

We evaluated changes in NV-HAP events among all patients admitted to 17 VA Medical Centers (1) across the period 10/01/2015–12/31/2019, and (2) one-year pre- vs post- each hospital’s oral care initiative start date. We modeled and compared observed versus predicted NV-HAP events per hospitalization using (1) an electronic clinical definition and (2) diagnosis codes, adjusting for patients’ demographics, vital signs, and laboratory results at presentation.

**Results:**

Among 333,257 hospitalizations, 1,922 (0.58%) met NV-HAP electronic clinical criteria and 2,386 (0.72%) diagnostic coding criteria. The risk of NV-HAP defined by electronic clinical criteria was 0.62% in October 2015 and 0.54% in December 2019 (estimated difference –0.084% [95% CI: –0.17%, 0.0056%]; the risk of NV-HAP defined by diagnostic coding decreased from 1.0% to 0.48% (estimated difference –0.53% [–0.63%, –0.43%]). In the one-year pre- vs post-analysis, there was no evidence of effect of the implementation on NV-HAP using either electronic clinical criteria (adjusted risk difference –0.078% (95% CI: –0.25%, 0.091%) or diagnostic coding criteria (adjusted risk difference –0.021% (95% CI: –0.18%, 0.14%).

**Conclusions:**

In a large multi-center study of hospitalized patients, we were unable to identify a clear effect of an oral care initiative on NV-HAP using electronic clinical criteria or diagnostic coding criteria.

## Introduction

Hospital-acquired pneumonia (HAP) is the most common healthcare-associated infection and is associated with high morbidity, mortality, and health care costs.^1^ Non-ventilator HAP (NV-HAP) accounts for the majority of HAP,^2^ but prevention efforts have historically focused on ventilator-associated pneumonia. NV-HAP is thought to be attributable to aspiration of oral microorganisms into the lungs, although the factors governing pathogenesis are poorly understood. Oral care, consisting of toothbrushing and denture cleaning, is a component of basic hospital care that may reduce NV-HAP risk and is listed as a recommended strategy for prevention.^3,4^ However, there are several barriers.^5–7^

After showing promise in reducing NV-HAP diagnoses in pilot sites and committing to the importance of oral care, the Veterans Affairs (VA) Administration implemented an oral care improvement initiative on a rolling basis starting in 2016.^8,9^ By July 2021, the initiative had been successfully adopted at every VA Medical Center in the nation as standard of care for all hospitalized patients and long-term residents and was recognized by the 2020 Gears of Government President’s Award for Innovation.^10^

Establishing the effectiveness of this initiative to prevent pneumonia, however, has been challenging, in part due to the lack of a reliable measure of NV-HAP.^11^ Surveillance definitions of NV-HAP include many subjective and ambiguous criteria that are difficult to apply consistently.^12^ Diagnosis codes have demonstrated poor sensitivity, specificity, and coding variability.^13^ Emerging evidence suggests that an electronic definition for NV-HAP based on changes in clinical parameters (oxygenation, temperature, white blood cell count, orders for chest imaging, and antibiotic administrations) can identify NV-HAP with higher accuracy than diagnosis codes and similar accuracy to traditional surveillance methods with less burden.^14–16^ In this study, we used both the electronic surveillance definition and diagnostic coding of NV-HAP to assess the impact of the oral care initiative on NV-HAP.

## Methods

### Settings and participants

**Figure 1. f1:**
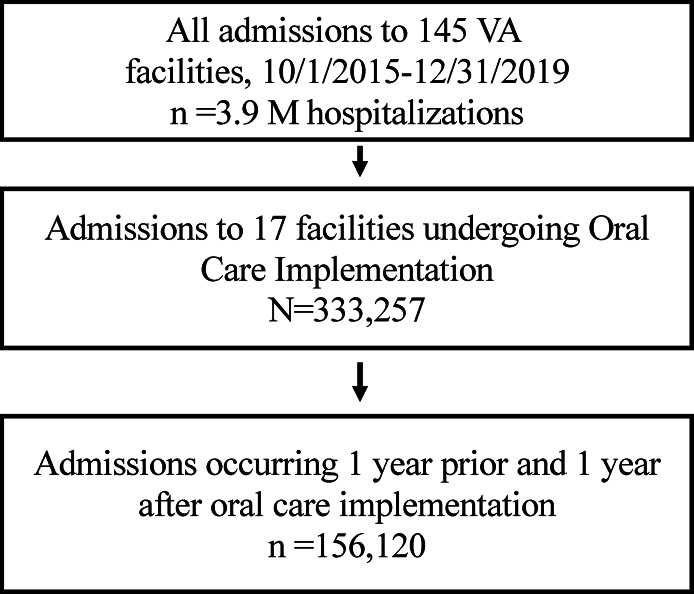
Study population.

The VA is the largest integrated healthcare system in the United States caring for 9 million Veterans nationwide.^17^ Clincial data were accessed through the Veterans Informatics and Computing Infrastructure (VINCI), a computing environment that stores clinical data for research purposes.^18^ We included only facilities that began implementing the oral care initiative at medical-surgical units at least one year before the COVID-19 pandemic; 17 facilities met these criteria. We extracted data for the period starting one year before the first facility began implementation through one year following the last facility’s implementation date, corresponding to January 10, 2015, through December 31, 2019 (Figure 1). The study was approved by VA and the University of Utah Institutional Review Boards.

### Measurements

#### Implementation of an oral care initiative

The VA’s oral care initiative aimed to standardize twice daily oral care for all patients hospitalized on medical-surgical and long-term care units through a well-planned, nurse-initiated implementation that followed evidence-based implementation principles informed by research.^19^ Full details have previously been described.^20^ In brief, the implementation contained 6 steps: (1) Prepare foundation (identify clinical champions, determine implementation approach, develop charter that outlines implementation, and engage stakeholders); (2) Obtain and organize supplies, including toothbrushes, toothpaste, and denture cleansing supplies; (3) Standardize templates, tools, and patient education materials; (4) Customize and conduct nursing staff trainings; (5) Implement; and (6) Monitor.^4^ We identified and verified the dates that each facility started and completed rolling out the initiative on medical-surgical wards.

#### Patient factors

We extracted patient demographics, comorbidities, vital signs, supplementary oxygen devices, laboratory tests, and discharge diagnosis codes. We defined and summarized comorbid conditions (Table 1) using the Charlson method applied to International Classification of Disease (ICD)-10-CM^21^ codes via software developed by the Agency for Healthcare Research and Quality.^22,23^

**Table 1.** Patient characteristics, risk of NV-HAP, and outcomes among (1) all patients hospitalized to 17 hospitals during January 1, 2015—December 31, 2019, and (2) patients hospitalized 1 year before the hospital start of oral care initiative, and (3) patients hospitalized 1 year after the start of oral care initiative

Characteristic	All hospitalizations		Hospitalizations in pre/post period		
Patient demographics	Missing	N = 333,257^b^	Overall, N = 156,120^b^	Before, N = 78,277^b^	After, N = 77,843^b^
Median (IQR) age^a^	0 (0%)	69 (61, 75)	69 (61, 75)	69 (61, 75)	69 (61, 75)
Sex^a^	0 (0%)				
Female		18,068 (5.4%)	8,462 (5.4%)	4,368 (5.6%)	4,094 (5.3%)
Male		315,189 (95%)	147,658 (95%)	73,909 (94%)	73,749 (95%)
Race	0 (0%)				
Asian		592 (0.2%)	288 (0.2%)	151 (0.2%)	137 (0.2%)
Black		88,437 (27%)	41,295 (26%)	20,840 (27%)	20,455 (26%)
Hispanic		39,637 (12%)	18,411 (12%)	9,091 (12%)	9,320 (12%)
White		193,911 (58%)	91,198 (58%)	45,653 (58%)	45,545 (59%)
Other/Missing		10,680 (3.2%)	4,928 (3.2%)	2,542 (3.2%)	2,386 (3.1%)
Admitting location	**0 (0%)**				
ICU		52,637 (16%)	24,880 (16%)	12,712 (16%)	12,168 (16%)
Observation		65,552 (20%)	29,001 (19%)	13,956 (18%)	15,045 (19%)
Ward		215,068 (65%)	102,239 (65%)	51,609 (66%)	50,630 (65%)
Admitting service	**0 (0%)**				
Cardiac surgery		4,377 (1.3%)	2,107 (1.3%)	1,166 (1.5%)	941 (1.2%)
Cardiology		4,523 (1.4%)	2,098 (1.3%)	1,092 (1.4%)	1,006 (1.3%)
Hospice		564 (0.2%)	290 (0.2%)	151 (0.2%)	139 (0.2%)
ICU		52,637 (16%)	24,880 (16%)	12,712 (16%)	12,168 (16%)
Medicine		149,262 (45%)	71,184 (46%)	35,939 (46%)	35,245 (45%)
Neurology		4,916 (1.5%)	2,391 (1.5%)	1,120 (1.4%)	1,271 (1.6%)
Oncology		3,519 (1.1%)	1,649 (1.1%)	723 (0.9%)	926 (1.2%)
Other		65,552 (20%)	29,001 (19%)	13,956 (18%)	15,045 (19%)
Surgery		47,907 (14%)	22,520 (14%)	11,418 (15%)	11,102 (14%)
Congestive heart failure	0 (0%)	90,257 (27%)	42,392 (27%)	21,398 (27%)	20,994 (27%)
Chronic lung disease	0 (0%)	110,928 (33%)	51,428 (33%)	25,383 (32%)	26,045 (33%)
Diabetes mellitus	0 (0%)	149,614 (45%)	70,107 (45%)	35,214 (45%)	34,893 (45%)
Chronic liver disease	0 (0%)	47,593 (14%)	22,578 (14%)	11,354 (15%)	11,224 (14%)
Cancer	0 (0%)	80,469 (24%)	37,079 (24%)	18,506 (24%)	18,573 (24%)
Neurological disease (dementia, hemiplegia)	0 (0%)	48,683 (15%)	23,283 (15%)	11,786 (15%)	11,497 (15%)
Chronic kidney disease	0 (0%)	112,025 (34%)	52,319 (34%)	26,537 (34%)	25,782 (33%)
Median (IQR) number of comorbidities	0 (0%)	3 (1, 4)	3 (1, 4)	3 (1, 4)	2 (1, 4)
Prior hospitalization (within 90 days)	0 (0%)	94,027 (28%)	43,850 (28%)	21,926 (28%)	21,924 (28%)
Physiologic measures					
Temperature < 36.0 or >38.0 C^a^	7,818 (2.3%)	45,974 (14%)	20,334 (13%)	10,018 (13%)	10,316 (14%)
Heart rate > 90 beats per minute^a^	6,911 (2.1%)	158,823 (49%)	74,841 (49%)	37,592 (49%)	37,249 (49%)
Respiratory rate > 20 breaths per minute^a^	8,386 (2.5%)	81,122 (25%)	37,606 (25%)	18,577 (24%)	19,029 (25%)
Systolic blood pressure<90 (mmHg)^a^	6,969 (2.1%)	18,845 (5.8%)	8,877 (5.8%)	4,408 (5.8%)	4,469 (5.9%)
Pulse oximetry <90% or on supplemental O2^a^	51,459 (15%)	58,284 (21%)	27,432 (21%)	13,453 (20%)	13,979 (21%)
Median (IQR) Body Mass Index^a^	14,339 (4.3%)	28 (24, 33)	28 (24, 33)	28 (24, 33)	28 (24, 33)
White blood cell count < 4 or > 12 K/uL^a^	52,080 (16%)	77,812 (28%)	36,736 (28%)	18,335 (28%)	18,401 (28%)
Blood urea nitrogen > 30 mg/dL^a^	50,609 (15%)	65,824 (23%)	30,894 (23%)	15,708 (24%)	15,186 (23%)
Creatinine > 2.0 g/dl^a^	50,288 (15%)	45,753 (16%)	21,295 (16%)	10,793 (16%)	10,502 (16%)
Glucose <60 or >250 mg/dL^a^	32,130 (9.6%)	47,099 (16%)	22,357 (16%)	11,266 (16%)	11,091 (16%)
Hematocrit < 30%^a^	48,675 (15%)	47,303 (17%)	22,069 (17%)	11,180 (17%)	10,889 (16%)
Sodium >130 mEq/L^a^	48,440 (15%)	14,647 (5.1%)	6,776 (5.1%)	3,522 (5.3%)	3,254 (4.9%)
Platelet count < 150 K/uL^a^	52,400 (16%)	51,716 (18%)	23,599 (18%)	11,571 (18%)	12,028 (18%)
Outcomes					
NV-HAP by electronic clinical criteria	0 (0%)	1,922 (0.6%)	864 (0.6%)	437 (0.6%)	427 (0.5%)
NV-HAP by diagnostic coding definition	0 (0%)	2,386 (0.7%)	1,100 (0.7%)	520 (0.7%)	580 (0.7%)
30-day mortality	0 (0%)	16,607 (5.0%)	8,012 (5.1%)	3,955 (5.1%)	4,057 (5.2%)
Hospital length-of-stay, median days	0 (0%)	4 (3, 7)	4 (3, 7)	4 (3, 7)	4 (3, 7)

a Included in adjusted analysis model.

b Median (IQR); n (%).

Laboratory values and vital signs within 2 calendar days prior to the hospitalization and on the first day of hospitalization were used and classified as “normal” or “abnormal” based on pre-defined cut-offs indicated in Table 1. Missing laboratories and vital signs were imputed as normal. We identified admitting specialty service and ward type (medical/surgical ward or intensive care unit). We also extracted facility characteristics. Thirty-day mortality was assessed using a vital status file maintained by the VA that includes data on deaths occurring both inside and outside of VA facilities.^24^

### Electronic clinical-data NV-HAP definition

The electronic clinical data definition was designed to identify non-ventilated patients with new respiratory deterioration (≥2 days of decreased oxygen saturation or increase in supplemental oxygen after ≥2 days of stable or improving oxygenation) and concurrent fever or leukocytosis, performance of chest imaging, and the administration of new antibiotics for at least 3 days.^14,16^ Complete details and SAS code describing the definition are publicly available at https://github.com/caramckenna/NVHAP/tree/main/.

### Diagnostic coding-based NV-HAP definition

We extracted a diagnostic coding-based NV-HAP definition previously used by the VA quality improvement initiative to track changes in NV-HAP risk.^25^ NV-HAP was defined as the presence of a primary or secondary discharge diagnosis code for pneumonia not present on admission (B95.3, B96.0, J13, J15.X, J16.X, J17.X, J18.X, J84.111, J84.116, J84.117, J84.2, J85.1, and J85.2).

### Statistical analysis

Among the entire population of hospitalizations at the 17 facilities during the study period, we calculated the percentage of hospitalizations with NV-HAP using both the electronic surveillance criteria and the diagnostic-coding-based criteria. For each hospitalization, only the first electronic surveillance event was counted.

### Temporal trends in NV-HAP, 2015–2019

We examined trends in each of the following outcomes:

1. NV-HAP[Fig f1], defined by:
  - The electronic clinical data criteria;
  - The diagnostic-coding based definition;
2. Death within 30 days of admission[Fig f2]; and
3. Length-of-stay[Fig f2].

We fit separate models for each outcome assuming a linear trend in days since the beginning of the study (10/01/2015). For NV-HAP, we compared the two definitions using a logistic regression model that included main effects for each definition, as well as an interaction term between definition and time; the interaction term was used to assess for difference in trends between the two definitions. The model was fit using generalized estimating equations (GEEs) to account for clustering by hospitalization. Logistic regression was used to model mortality, and a Hurdle model (described below)^26^ was used for length-of-stay. To visualize trends over time, we plotted the estimated percentages for NV-HAP and mortality and means for length-of-stay along with the observed percentages/means by quarter.

### Effect of oral care implementation on NV-HAP (1-year pre- versus 1-year post-implementation)

#### Unadjusted analysis

For each site, we calculated crude differences in the following outcomes 1-year post-implementation versus 1-year pre-implementation: the percentage of hospitalizations with ≥1 NV-HAP event according to the electronic surveillance definition and according to the diagnostic coding definition; the percentage of hospitalizations that culminated in death within 30 days of admission; and mean length-of-stay.

#### Adjusted analysis

For each definition, we sought to compare, on a risk difference scale, the *counterfactual* risk of hospitalization with an NV-HAP event for the year post-implementation if, contrary to fact, there had been no implementation, to the *actual* risk of hospitalization with an NV-HAP event for the year post-implementation. Our approach accounts for site-level variability in risk differences and the possibility that the intervention could have a direct effect on preventing NV-HAP, and/or an indirect effect on NV-HAP through changing length of stay.

We estimated the counterfactual risk as follows. Using data one year before implementation from the 17 sites, we fit models for (1) the conditional distribution of length-of-stay given site, calendar year and week of hospitalization, patient characteristics, and health status variables available through day of admission and (2) the conditional probability of NV-HAP given length-of-stay and the same factors included in the length-of-stay model. Specific patient variables used are listed in Table 1. A hurdle model^26^ with a negative binomial count component was specified for (1), and a logistic regression model was specified for (2). The estimated fits of these models were used to compute, for each patient admitted to one of the 17 sites during the post-implementation period and every possible level of length-of-stay (*l* = 1,2, …), (a) the counterfactual probability of length-of-stay level *l* and (b) the counterfactual probability of NV-HAP given length-of-stay level *l*. All these quantities were combined, using the law of total probability, to estimate, for each patient, the counterfactual probability of NV-HAP. These estimated probabilities were then averaged over patients at each site to create site-specific counterfactual estimated risks. For each site, we computed the actual risk of NV-HAP events per hospitalization during the post-implementation period. Each site’s risk difference was then estimated as the difference between the site-specific estimated counterfactual and actual risks.

#### Pooled analyses

For both the unadjusted and adjusted analyses, the variance-covariance matrix of the site-specific risk differences was estimated using the delta method.^27^ A pooled estimate of site-specific effects was computed using meta-analysis with inverse-variance weighting of site-specific estimates. Computation of the standard error of the pooled estimate accounted for the correlation of the site-specific estimates.

#### Secondary analyses

We used the same approach to estimate the effect of implementation on 30-day mortality and hospital length-of-stay using logistic regression and hurdle models, respectively. We also conducted subgroup analyses restricted to patients admitted only to non-ICU wards (the primary target of implementation), with implementation dates shifted to 3 months prior to the listed start date (to account for anticipatory effects) as well as shifted to the listed completion dates of the implementation (to account for delayed effects). All statistical analyses were performed using RStudio (version 1.4; RStudio, PBC, Boston, MA, 2021).

## Results

### Descriptive characteristics

There were 333,257 patient hospitalizations during the study period. Of these, 156,120 occurred within 1 year before or after their facility’s implementation start (Figure 1). Median age was 69 (interquartile range 61, 75), 95% were male, and 16% were admitted to intensive care units. Median time to completing roll-out of the implementation at all medical/surgical wards was 8 months (intra-quartile range: 6–12 months). Patient characteristics and illness severity (Table 1) were similar between pre- and post-implementation periods as well the national VA population.^14^

### Temporal trends in NV-HAP, mortality, and length of stay (2015–2019)

**Figure 2. f2:**
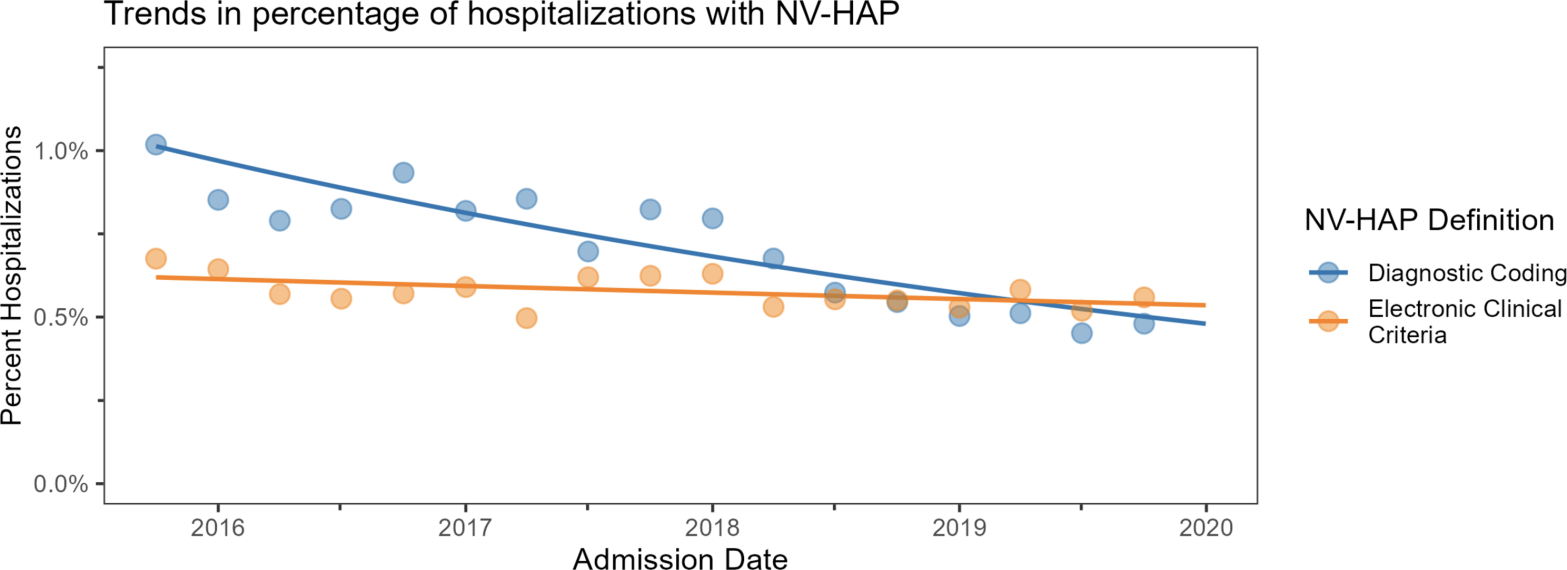
Trends in NV-HAP risk by electronic surveillance versus claims-based definition.

Among 333,257 hospitalizations, 1,922 (0.58%) met NV-HAP clinical surveillance criteria, and 2,386 (0.72%) met diagnostic coding criteria. Trends in NV-HAP across the study period are shown in Figure 2. Using the electronic surveillance definition, the percentage of hospitalizations with NV-HAP was 0.62% in October 2015 and 0.54% in Dec 2019 (estimated difference –0.084% [95% CI: –0.17%, 0.0056%]; estimated log odds change per year –0.034% [95% CI: –0.071%, 0.0023%]). Using the diagnostic coding definition, NV-HAP decreased from 1.0% to 0.48%. (estimated difference –0.53% [95% CI: –0.63%, –0.43%]; estimated log odds change per year –0.18, [95% CI: –0.21, –0.15]). The difference in the estimated log-odds change per year measured by electronic surveillance coding versus diagnostic coding was 0.14 (95% CI: 0.097, 0.19).

**Figure 3.**
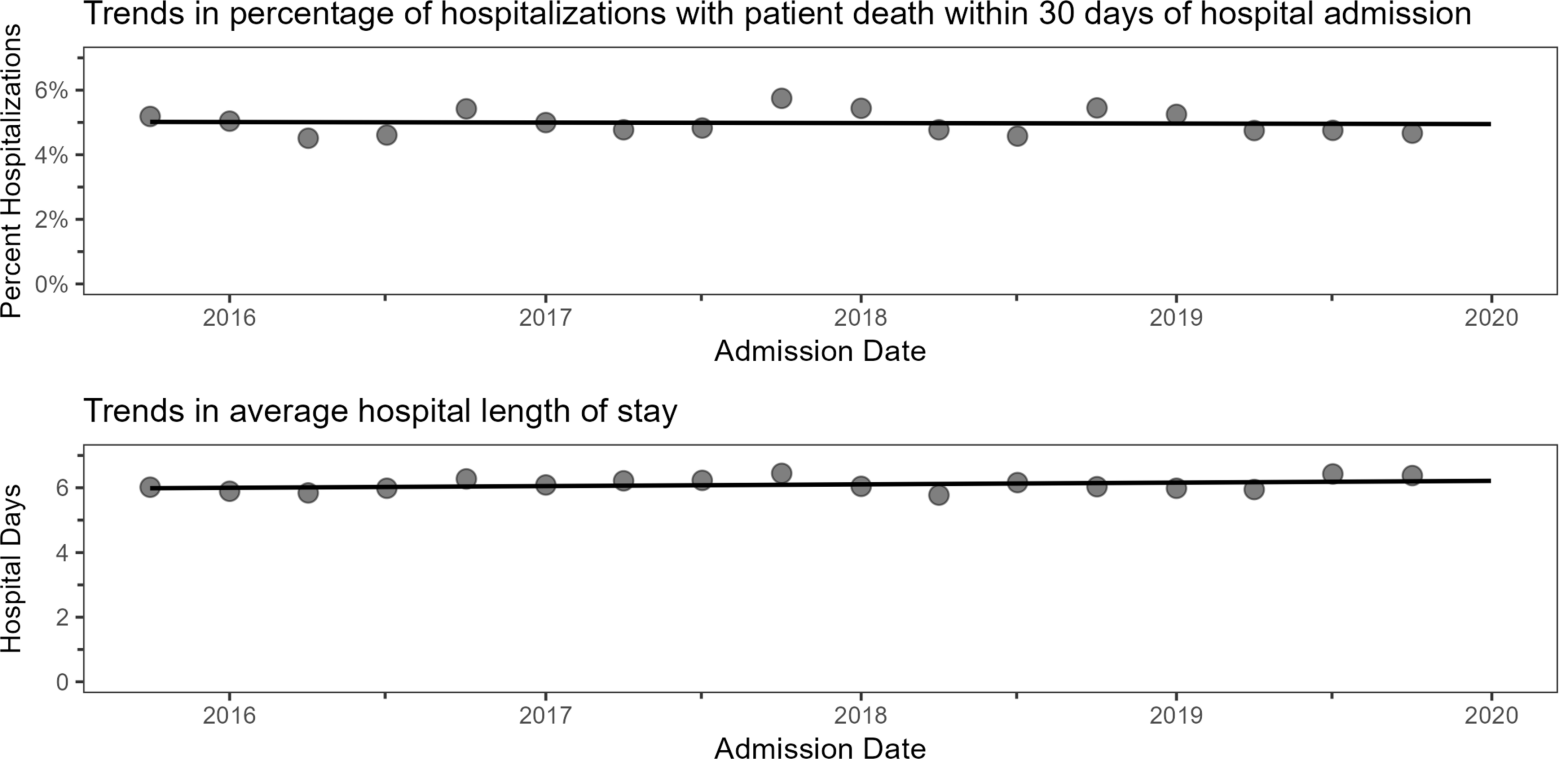
Trends in 30-day mortality and hospital length of stay.

30-day mortality among all hospitalizations was 4.9%, which fluctuated by season and year without an appreciable trend over time (estimated probability of 30-day mortality 5.02% in 2015 to 4.95% in 2019 (estimated difference –0.066% [95% CI: –0.32%, 0.19%] (Figure 3A). The overall estimated mean length-of-stay was 6.1 days. This fluctuated by season with a net increase across the study period from 5.98 days to 6.21 days (estimated difference 0.23 days [95% CI: 0.16, 0.30]) (Figure 3B).

### Effect of oral care implementation on NV-HAP (1-year pre- vs post-implementation)

Among the 156,120 hospitalizations in the pre-post analysis, 77,300 occurred within the year pre-implementation and 78,296 in the year post-implementation. Changes in NV-HAP, mortality and length-of-stay before and after each facility’s implementation dates are shown in eFigures 2–4. Using the electronic clinical surveillance definition, 427 (0.55%) had NV-HAP in the year before implementation versus 437 (0.56%) in the year after implementation. Using the diagnostic coding definition, 580 (0.75%) had NV-HAP in the year before implementation versus 520 (0.66%) in the year after.

**Figure 4.**
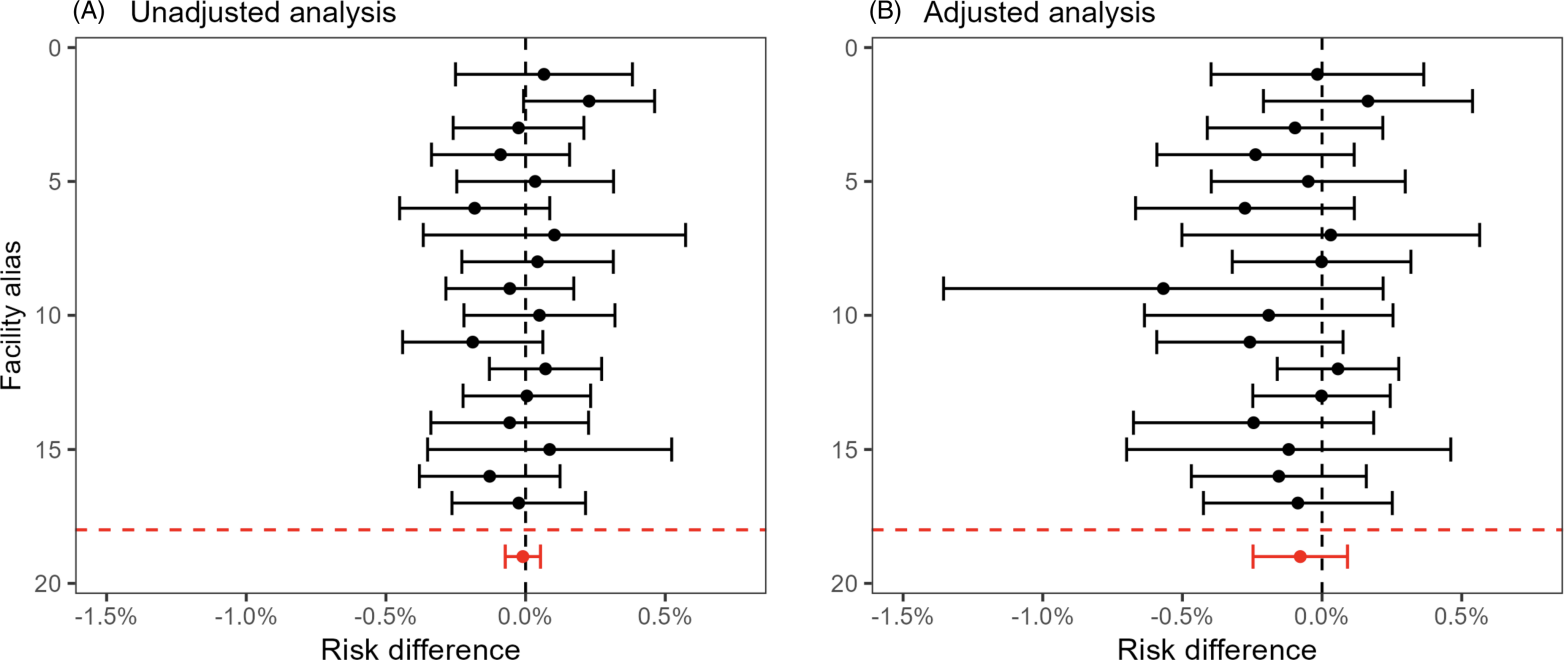
Effect of oral care implementation on NV-HAP risk defined by electronic clinical criteria. Panel A. Unadjusted analysis: differences in percentage of NV-HAP events 1 year post versus 1 year prior to implementation among 17 facilities undergoing oral care implementation. Panel B. Adjusted analysis: differences in observed percentage of NV-HAP events post implementation versus predicted probability after accounting for patient characteristics.

**Figure 5.**
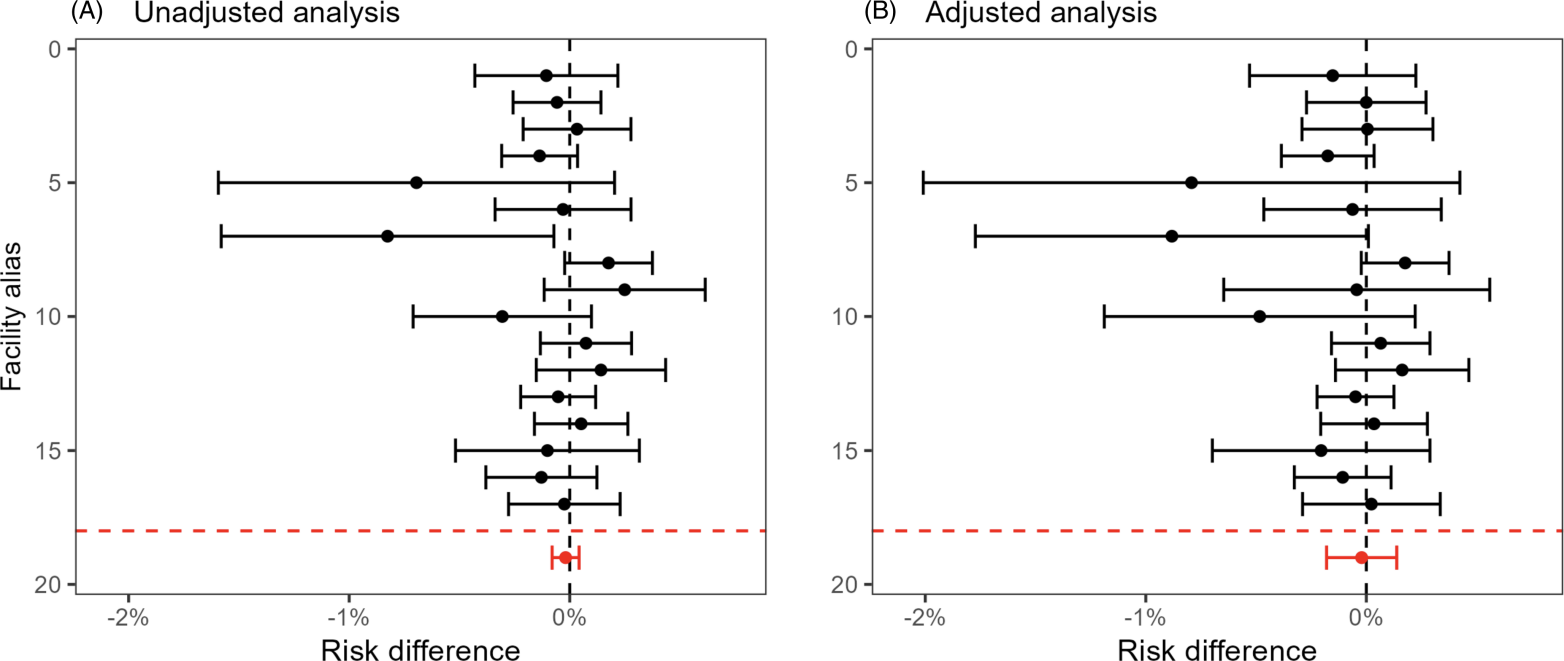
Effect of oral care implementation on NV-HAP defined by diagnostic coding. Panel A. Unadjusted analysis: differences in proportion of NV-HAP diagnoses 1 year post versus 1 year prior to implementation among 17 facilities undergoing oral care implementation. Panel B. Adjusted analysis: Differences in observed proportion of NV-HAP diagnoses post-implementation versus predicted probability after accounting for patient characteristics.

**Figure 6.**
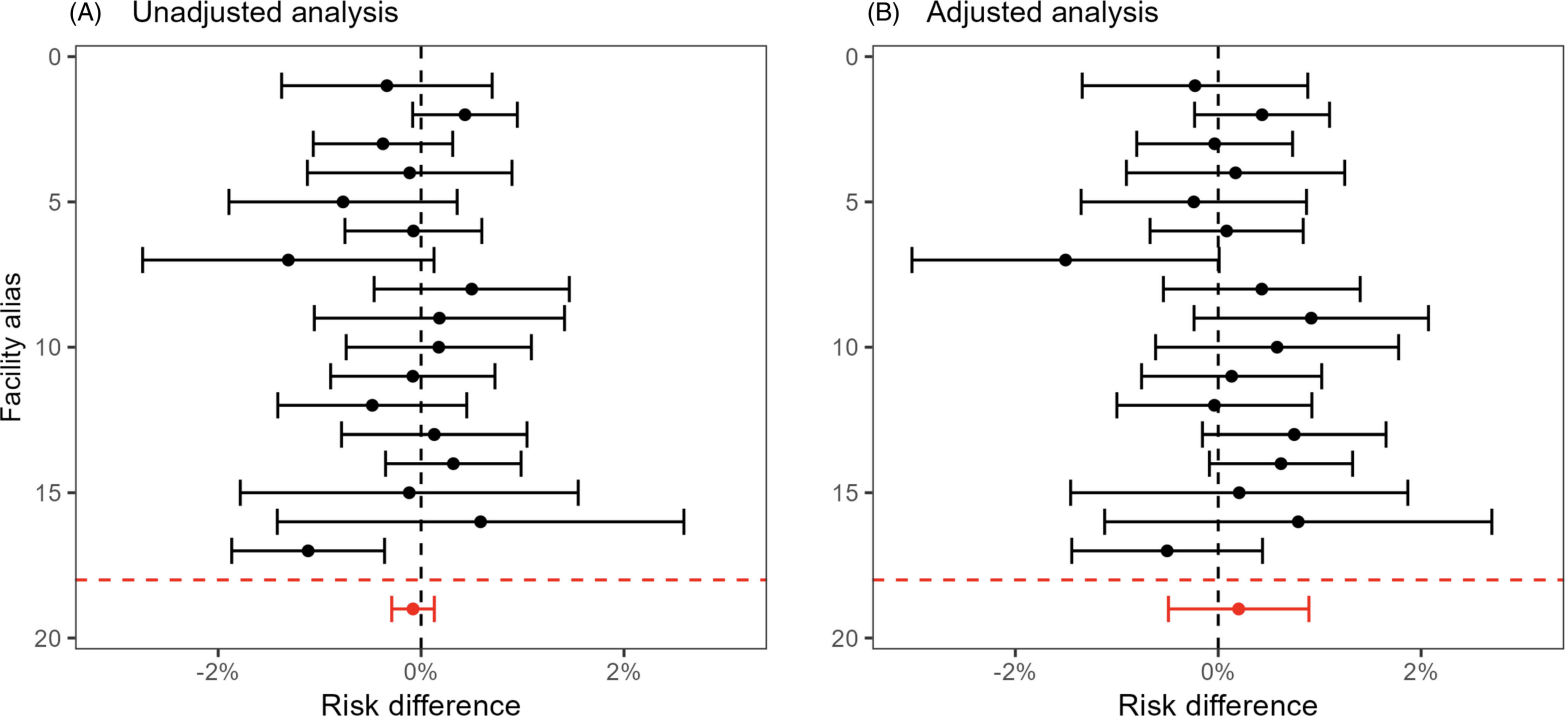
Effect of oral care implementation on 30-day mortality. Panel A. Unadjusted analysis: differences in proportion of NV-HAP events 1 year post versus 1 year prior to implementation among 17 facilities undergoing oral care implementation. Panel B. Adjusted analysis: Differences in observed proportion of 30-day mortality events post implementation versus predicted probability after accounting for patient characteristics.

Figure 4 (electronic surveillance) and Figure 5 (diagnostic coding) present the unadjusted (left panels) and adjusted (right panels) site-specific and overall estimated risk differences and confidence intervals. The overall adjusted risk differences were –0.078% (95% CI: –0.25%, 0.091%) and –0.021% (95% CI: –0.18%, 0.14%) for the electronic surveillance and diagnostic coding criteria, respectively. Thus, there was no evidence of an effect of the oral care initiative on NV-HAP using either definition. There was also no detectable effect on 30-day mortality (Figure 6), with an adjusted risk difference of 0.20% (95% CI: –0.49%, 0.89%). The adjusted mean difference in length-of-stay (eFigure 1) was –0.13 days (95% CI: –0.24 days, –0.02 days).

The results were similar for all secondary analyses (Supplement eFigures 5–10). For the subgroup of patients admitted to non-ICU wards, NV-HAP rates did not change appreciably using either the electronic surveillance or diagnostic coding criteria. When the implementation date was shifted 3 months earlier than the formal start date or to 3 months after the completion date, there was no detectable effect on NV-HAP or mortality, but a small effect on length-of-stay.

## Discussion

In a large population of patients hospitalized at 17 VA hospitals that implemented an oral care initiative on at least one medical surgical unit between 2015 and 2019, there was a temporal decrease in NV-HAP defined by diagnostic coding but not when using electronic clinical criteria. There was no detectable difference in NV-HAP rates in the year before vs after implementation using either diagnostic code criteria or electronic clinical criteria, no detectable effect on 30-day mortality, and a small effect on length-of-stay.

The temporal decrease in NV-HAP defined by diagnostic coding is consistent with a previous system-wide evaluation of the VA that found a 32% decrease in NV-HAP diagnoses between 2015 and 2020.^25^ However, we did not observe such a dramatic trend using electronic clinical criteria for NV-HAP. Since a large CDC point-prevalence study^28^ documented the high burden of HAP between 2011 and 2015, multiple efforts to measure and reduce NV-HAP were launched, including an updated guideline for prevention.^4^ Other studies have reported inconsistent trends during this period: Baker et al reported a temporal increase in NV-HAP-associated costs for Medicaid beneficiaries using diagnosis codes between 2015 and 2019,^29^ and a study using the Medicare Patient Monitoring System reported a decrease in post-operative pneumonia, which could suggest that efforts to prevent NV-HAP may be having an effect.^30^ Our results were limited to 17 facilities; so, a larger analysis may show a clearer system-wide improvement across the entire VA. The difference in trends using diagnosis codes versus electronic clinical criteria may reflect the subjectivity and variable usage of codes by different hospitals.^13,31^ However, the initiative did not appear to have a detectable effect on NV-HAP diagnoses at the facility level, which suggests that implementation of the initiative did not directly result in any local coding shifts.

Our failure to identify an effect of oral care implementation on NV-HAP builds on existing evidence on oral care implementation, which has been inconsistent. One cluster randomized trial reported reduction in NV-HAP with an oral care protocol, although the difference was significant for surgical but not medical patients. Three prior pre-post implementation studies reported decreases in NV-HAP diagnoses with oral care initiatives in acute settings,^3,32–35^ and one non-randomized comparative study did not.^36^ Two clinical trials of nursing home patients demonstrated negative results.^37,38^

There are several potential explanations for our findings. First, improvements in NV-HAP from the initiative could have evaded our measurement strategies and observational study design. While we evaluated the initiative using two definitions of NV-HAP, both are imperfect measures of rare events, and pneumonia remains difficult to accurately assess due to its lack of gold standard criteria. However, the lack of substantial reductions in mortality or length-of-stay—two objective measures that should be impacted by reductions in NV-HAP—make it less likely that we missed a large positive finding due to measurement error. Our retrospective analysis of observational data allows for the possibility of residual confounding, despite the breadth of variables included in our adjusted analyses.

Second, the initiative as we captured it may not have been effective at increasing oral care, either due to low fidelity of the initiative, complexity of the implementation, or high baseline adherence to oral care in the studied hospitals. We were unable to directly assess oral care provided in study hospitals, making it difficult to differentiate these possibilities. Oral care implementation was targeted in a rolling fashion to all medical-surgical units, while our primary analysis evaluated all patients hospitalized in all units within each facility (most of which were medical-surgical units) which could have missed isolated decreases in NV-HAP within target units. Our primary analysis was also based upon a discrete start date per hospital, while implementations were phased, with a median time to completing roll-out of 8 months; so, the effect of the initiative may have lagged the evaluation period for some hospitals. However, secondary analyses that evaluated only non-ICU patients and that shifted implementation dates to account for anticipatory or delayed effects of phased implementation yielded similar results. The oral care initiative was also conducted in parallel with national efforts to raise awareness of NV-HAP and the importance of oral care throughout the VA system.^25^ It is thus possible that, VA’s national commitment to NV-HAP prevention in 2016 catalyzed nurses to improve oral care prior to the initiative. This may explain the system-level temporal trends but lack of facility-level effect.

Third, enhanced oral care alone may not be sufficient to prevent NV-HAP. The pathogenesis of pneumonia is complex, with multiple contributing factors including the burden of oral flora, the frequency and quantity of aspiration, host airway clearance capacity, and host immunity. Targeting multiple processes simultaneously including patient mobility, dysphagia management, respiratory therapy, minimizing sedation, and delirium prevention may be necessary to prevent this condition.^4,11^ A recent clinical trial by Wolfensberger et al demonstrated a correlation between effective implementation of such an intervention and a reduction of NV-HAP, highlighting the importance of multimodal strategies.^39^

Our results do not reduce the importance of oral care to patient comfort, oral health, and hospital quality. Its benefits extend well beyond pneumonia prevention alone. Maintaining oral health is a fundamental component of physical and personal well-being and impacts nutrition, communication, comfort, and quality of life,^40^ yet poor oral health is common among hospitalized patients.^41^ Promoting oral health during hospitalizations not only reduces hospital complications but also promotes oral health upon discharge.^42^ Despite its importance, oral care is difficult to prioritize without standardization and support.^43^ Oral care initiatives such as the one implemented across the VA system are thus essential components of high-quality hospital care in addition to pneumonia prevention efforts.

Our analysis adds to existing evidence regarding the complexity of NV-HAP development, prevention, and measurement that challenge the evaluation of strategies for prevention. Our study suggests that initiatives targeting oral care alone may not be sufficient to reduce NV-HAP rates. These results stress the need to evaluate multimodal, complex interventions, and use objective clinical measures to advance our understanding of NV-HAP pathogenesis and prevention. Large studies that evaluate the impact of multiple prevention strategies on multiple outcomes—including objective definitions of NV-HAP as well as length of stay, mortality, microbiologic findings, and key patient-centered outcomes—are needed to advance understanding of the pathogenesis of NV-HAP and identify which strategies prevent it.

## Supporting information

Jones et al. supplementary material Jones et al. supplementary material

## Supplementary material

The supplementary material for this article can be found at https://doi.org/10.1017/ice.2025.54
