# Supplementary material for: Evaluating the impact of an oral care initiative on the risk of non-ventilator-associated hospital-acquired pneumonia using electronic clinical data and diagnostic coding surveillance criteria
Source: Infect Control Hosp Epidemiol. 2025 Oct 15;46(12):1190–8. doi: 10.1017/ice.2025.54 (PMC12779461; doi:10.1017/ice.2025.54)
Supplement: Jones et al. supplementary material [file S0899823X25000546sup001.docx]

Supplementary Tables.

## eTable 1. First versus last 4 quarters of study period. * indicates patient factors used in adjusted analyses.

|  | **All hospitalizations** | | **Hospitalizations in first 4 quarters vs last 4 quarters** | | |
| --- | --- | --- | --- | --- | --- |
| **Characteristic** | **Missing** | **N = 333,257**^1^ | **Overall**, N = 158,473^1^ | **First 4 Quarters**, N = 80,817^1^ | **Last 4 Quarters**, N = 77,656^1^ |
| Median (IQR) age* | 0 (0%) | 69 (61, 75) | 69 (61, 75) | 68 (61, 75) | 70 (62, 76) |
| Gender* | 0 (0%) |  |  |  |  |
| Female |  | 18,068 (5.4%) | 8,726 (5.5%) | 4,285 (5.3%) | 4,441 (5.7%) |
| Male |  | 315,189 (95%) | 149,747 (94%) | 76,532 (95%) | 73,215 (94%) |
| Race | 0 (0%) |  |  |  |  |
| Asian |  | 592 (0.2%) | 275 (0.2%) | 131 (0.2%) | 144 (0.2%) |
| Black |  | 88,437 (27%) | 42,372 (27%) | 21,081 (26%) | 21,291 (27%) |
| Hispanic |  | 39,637 (12%) | 18,878 (12%) | 9,636 (12%) | 9,242 (12%) |
| White |  | 193,911 (58%) | 91,797 (58%) | 47,405 (59%) | 44,392 (57%) |
| Other/Missing |  | 10,680 (3.2%) | 5,151 (3.3%) | 2,564 (3.2%) | 2,587 (3.3%) |
| Congestive heart failure | 0 (0%) | 90,257 (27%) | 43,076 (27%) | 20,966 (26%) | 22,110 (28%) |
| Chronic lung disease | 0 (0%) | 110,928 (33%) | 53,099 (34%) | 27,622 (34%) | 25,477 (33%) |
| Diabetes mellitus | 0 (0%) | 149,614 (45%) | 71,284 (45%) | 35,650 (44%) | 35,634 (46%) |
| Chronic liver disease | 0 (0%) | 47,593 (14%) | 22,541 (14%) | 11,104 (14%) | 11,437 (15%) |
| Cancer | 0 (0%) | 80,469 (24%) | 38,834 (25%) | 19,868 (25%) | 18,966 (24%) |
| Neurological disease (dementia, hemiplegia) | 0 (0%) | 48,683 (15%) | 22,487 (14%) | 10,702 (13%) | 11,785 (15%) |
| Chronic kidney disease | 0 (0%) | 112,025 (34%) | 53,720 (34%) | 26,196 (32%) | 27,524 (35%) |
| Median (IQR) number of comorbidities | 0 (0%) | 3 (1, 4) | 3 (1, 4) | 2 (1, 4) | 3 (1, 4) |
| Prior hospitalization (within 90 days) | 0 (0%) | 94,027 (28%) | 45,156 (28%) | 23,153 (29%) | 22,003 (28%) |
| White blood cell count < 4 or > 12 K/uL* | 52,080 (16%) | 77,812 (28%) | 36,664 (27%) | 18,523 (27%) | 18,141 (27%) |
| Blood urea nitrogen > 30 mg/dL* | 50,609 (15%) | 65,824 (23%) | 31,263 (23%) | 14,996 (22%) | 16,267 (24%) |
| Creatinine > 2.0 g/dl* | 50,288 (15%) | 45,753 (16%) | 22,031 (16%) | 10,873 (16%) | 11,158 (17%) |
| Glucose <60 or >250 mg/dL* | 32,130 (9.6%) | 47,099 (16%) | 22,249 (16%) | 10,947 (15%) | 11,302 (16%) |
| Hematocrit < 30%* | 48,675 (15%) | 47,303 (17%) | 22,099 (16%) | 10,830 (16%) | 11,269 (17%) |
| Sodium >130 mEq/L* | 48,440 (15%) | 14,647 (5.1%) | 7,077 (5.2%) | 3,573 (5.2%) | 3,504 (5.2%) |
| Platelet count < 150 K/uL* | 52,400 (16%) | 51,716 (18%) | 24,517 (18%) | 13,218 (20%) | 11,299 (17%) |
| Temperature < 36.0 or >38.0 C* | 7,818 (2.3%) | 45,974 (14%) | 22,322 (14%) | 12,547 (16%) | 9,775 (13%) |
| Heart rate > 90 beats per minute* | 6,911 (2.1%) | 158,823 (49%) | 74,746 (48%) | 37,646 (48%) | 37,100 (49%) |
| Respiratory rate > 20 breaths per minute* | 8,386 (2.5%) | 81,122 (25%) | 38,485 (25%) | 20,031 (25%) | 18,454 (24%) |
| Systolic blood pressure<90 (mmHg)* | 6,969 (2.1%) | 18,845 (5.8%) | 8,848 (5.7%) | 4,439 (5.6%) | 4,409 (5.8%) |
| Pulse oximetry <90% or on supplemental O2* | 51,459 (15%) | 58,284 (21%) | 27,054 (20%) | 14,621 (21%) | 12,433 (19%) |
| Median (IQR) Body Mass Index* | 14,339 (4.3%) | 28 (24, 33) | 28 (24, 33) | 28 (24, 33) | 28 (24, 33) |
| **Admitting location** |  |  |  |  |  |
| ICU |  | 52,637 (16%) | 24,849 (16%) | 12,432 (15%) | 12,417 (16%) |
| Observation |  | 65,552 (20%) | 32,824 (21%) | 17,209 (21%) | 15,615 (20%) |
| Ward |  | 215,068 (65%) | 100,800 (64%) | 51,176 (63%) | 49,624 (64%) |
| **Outcomes** |  |  |  |  |  |
| NV-HAP by electronic surveillance criteria | 0 (0%) | 1,922 (0.6%) | 919 (0.6%) | 494 (0.6%) | 425 (0.5%) |
| NV-HAP by coding/claims definition | 0 (0%) | 2,386 (0.7%) | 1,082 (0.7%) | 704 (0.9%) | 378 (0.5%) |
| 30-day mortality | 0 (0%) | 16,607 (5.0%) | 7,682 (4.8%) | 3,910 (4.8%) | 3,772 (4.9%) |
| Hospital length-of-stay, median days | 0 (0%) | 4 (3, 7) | 4 (3, 7) | 4 (3, 7) | 4 (3, 7) |
| ^1^Median (IQR); n (%) | | | | | |

## eTable 2. Non-ICU admissions. * indicates patient factors used in adjusted analyses.

|  | **Hospitalizations 1 year before and after implementation** | | **Missingness** | |
| --- | --- | --- | --- | --- |
| **Characteristic** | **0**, N = 77,843^1^ | **1**, N = 78,277^1^ | **0**, N = 77,843^2^ | **1**, N = 78,277^2^ |
| Median (IQR) age* | 69 (61, 75) | 69 (61, 75) | 0 (0%) | 0 (0%) |
| Gender* |  |  |  |  |
| Female | 4,094 (5.3%) | 4,368 (5.6%) | 0 (0%) | 0 (0%) |
| Male | 73,749 (95%) | 73,909 (94%) | 0 (0%) | 0 (0%) |
| Race |  |  |  |  |
| Asian | 137 (0.2%) | 151 (0.2%) | 0 (0%) | 0 (0%) |
| Black | 20,455 (26%) | 20,840 (27%) | 0 (0%) | 0 (0%) |
| Hispanic | 9,320 (12%) | 9,091 (12%) | 0 (0%) | 0 (0%) |
| White | 45,545 (59%) | 45,653 (58%) | 0 (0%) | 0 (0%) |
| Other/Missing | 2,386 (3.1%) | 2,542 (3.2%) | 0 (0%) | 0 (0%) |
| Congestive heart failure | 20,994 (27%) | 21,398 (27%) | 0 (0%) | 0 (0%) |
| Chronic lung disease | 26,045 (33%) | 25,383 (32%) | 0 (0%) | 0 (0%) |
| Diabetes mellitus | 34,893 (45%) | 35,214 (45%) | 0 (0%) | 0 (0%) |
| Chronic liver disease | 11,224 (14%) | 11,354 (15%) | 0 (0%) | 0 (0%) |
| Cancer | 18,573 (24%) | 18,506 (24%) | 0 (0%) | 0 (0%) |
| Neurological disease (dementia, hemiplegia) | 11,497 (15%) | 11,786 (15%) | 0 (0%) | 0 (0%) |
| Chronic kidney disease | 25,782 (33%) | 26,537 (34%) | 0 (0%) | 0 (0%) |
| Median (IQR) number of comorbidities | 2 (1, 4) | 3 (1, 4) | 0 (0%) | 0 (0%) |
| Prior hospitalization (within 90 days) | 21,924 (28%) | 21,926 (28%) | 0 (0%) | 0 (0%) |
| White blood cell count < 4 or > 12 K/uL* | 18,401 (24%) | 18,335 (23%) | 12,258 (16%) | 12,249 (16%) |
| Blood urea nitrogen > 30 mg/dL* | 15,186 (20%) | 15,708 (20%) | 11,974 (15%) | 11,667 (15%) |
| Creatinine > 2.0 g/dl* | 10,502 (13%) | 10,793 (14%) | 11,880 (15%) | 11,607 (15%) |
| Glucose <60 or >250 mg/dL* | 11,091 (14%) | 11,266 (14%) | 7,512 (9.7%) | 7,371 (9.4%) |
| Hematocrit < 30%* | 10,889 (14%) | 11,180 (14%) | 11,456 (15%) | 11,468 (15%) |
| Sodium >130 mEq/L* | 3,254 (4.2%) | 3,522 (4.5%) | 11,425 (15%) | 11,198 (14%) |
| Platelet count < 150 K/uL* | 12,028 (15%) | 11,571 (15%) | 12,316 (16%) | 12,362 (16%) |
| Temperature < 36.0 or >38.0 C* | 10,316 (13%) | 10,018 (13%) | 1,694 (2.2%) | 1,977 (2.5%) |
| Heart rate > 90 beats per minute* | 37,249 (48%) | 37,592 (48%) | 1,531 (2.0%) | 1,786 (2.3%) |
| Respiratory rate > 20 breaths per minute* | 19,029 (24%) | 18,577 (24%) | 1,892 (2.4%) | 2,139 (2.7%) |
| Systolic blood pressure<90 (mmHg)* | 4,469 (5.7%) | 4,408 (5.6%) | 1,553 (2.0%) | 1,801 (2.3%) |
| Pulse oximetry <90% or on supplemental O2* | 13,979 (18%) | 13,453 (17%) | 11,746 (15%) | 12,171 (16%) |
| Median (IQR) Body Mass Index* | 28 (24, 33) | 28 (24, 33) | 3,455 (4.4%) | 3,201 (4.1%) |
| **Admitting location** |  |  |  |  |
| ICU | 12,168 (16%) | 12,712 (16%) | 0 (0%) | 0 (0%) |
| Observation | 15,045 (19%) | 13,956 (18%) | 0 (0%) | 0 (0%) |
| Ward | 50,630 (65%) | 51,609 (66%) | 0 (0%) | 0 (0%) |
| **Outcomes** |  |  |  |  |
| NV-HAP by electronic surveillance criteria | 427.00 (0.55%) | 437.00 (0.56%) | 0 (0%) | 0 (0%) |
| NV-HAP by coding/claims definition | 580.00 (0.75%) | 520.00 (0.66%) | 0 (0%) | 0 (0%) |
| 30-day mortality | 4,057 (5.2%) | 3,955 (5.1%) | 0 (0%) | 0 (0%) |
| Median (IQR) and mean (SD) hospital length-of-stay |  |  | 0 (0%) | 0 (0%) |
| Median (IQR) | 4 (3, 7) | 4 (3, 7) |  |  |
| Mean (SD) | 6 (15) | 6 (17) |  |  |
| ^1^Median (IQR); n (%) | | | | |
| ^2^N missing (% missing); N missing (% missing) | | | | |

## eTable 3. Secondary Analysis - Shifted Implementation Date

|  | **All hospitalizations** | | **Hospitalizations in pre/post period with shifted implementation date** | | |
| --- | --- | --- | --- | --- | --- |
| **Characteristic** | **Missing** | **N = 333,257**^1^ | **Overall**, N = 155,596^1^ | **0**, N = 77,300^1^ | **1**, N = 78,296^1^ |
| Median (IQR) age* | 0 (0%) | 69 (61, 75) | 69 (61, 75) | 68 (61, 75) | 69 (61, 75) |
| Gender* | 0 (0%) |  |  |  |  |
| Female |  | 18,068 (5.4%) | 8,316 (5.3%) | 3,899 (5.0%) | 4,417 (5.6%) |
| Male |  | 315,189 (95%) | 147,280 (95%) | 73,401 (95%) | 73,879 (94%) |
| Race | 0 (0%) |  |  |  |  |
| Asian |  | 592 (0.2%) | 270 (0.2%) | 123 (0.2%) | 147 (0.2%) |
| Black |  | 88,437 (27%) | 41,014 (26%) | 20,232 (26%) | 20,782 (27%) |
| Hispanic |  | 39,637 (12%) | 18,592 (12%) | 9,339 (12%) | 9,253 (12%) |
| White |  | 193,911 (58%) | 90,803 (58%) | 45,146 (58%) | 45,657 (58%) |
| Other/Missing |  | 10,680 (3.2%) | 4,917 (3.2%) | 2,460 (3.2%) | 2,457 (3.1%) |
| Congestive heart failure | 0 (0%) | 90,257 (27%) | 41,905 (27%) | 20,720 (27%) | 21,185 (27%) |
| Chronic lung disease | 0 (0%) | 110,928 (33%) | 51,468 (33%) | 26,014 (34%) | 25,454 (33%) |
| Diabetes mellitus | 0 (0%) | 149,614 (45%) | 69,737 (45%) | 34,669 (45%) | 35,068 (45%) |
| Chronic liver disease | 0 (0%) | 47,593 (14%) | 22,429 (14%) | 11,018 (14%) | 11,411 (15%) |
| Cancer | 0 (0%) | 80,469 (24%) | 36,787 (24%) | 18,396 (24%) | 18,391 (23%) |
| Neurological disease (dementia, hemiplegia) | 0 (0%) | 48,683 (15%) | 23,204 (15%) | 11,263 (15%) | 11,941 (15%) |
| Chronic kidney disease | 0 (0%) | 112,025 (34%) | 51,643 (33%) | 25,413 (33%) | 26,230 (34%) |
| Median (IQR) number of comorbidities | 0 (0%) | 3 (1, 4) | 2 (1, 4) | 2 (1, 4) | 3 (1, 4) |
| Prior hospitalization (within 90 days) | 0 (0%) | 94,027 (28%) | 43,665 (28%) | 21,793 (28%) | 21,872 (28%) |
| White blood cell count < 4 or > 12 K/uL* | 52,080 (16%) | 77,812 (28%) | 36,728 (28%) | 18,302 (28%) | 18,426 (28%) |
| Blood urea nitrogen > 30 mg/dL* | 50,609 (15%) | 65,824 (23%) | 30,524 (23%) | 14,969 (23%) | 15,555 (23%) |
| Creatinine > 2.0 g/dl* | 50,288 (15%) | 45,753 (16%) | 21,089 (16%) | 10,381 (16%) | 10,708 (16%) |
| Glucose <60 or >250 mg/dL* | 32,130 (9.6%) | 47,099 (16%) | 22,264 (16%) | 11,065 (16%) | 11,199 (16%) |
| Hematocrit < 30%* | 48,675 (15%) | 47,303 (17%) | 21,967 (17%) | 10,887 (17%) | 11,080 (17%) |
| Sodium >130 mEq/L* | 48,440 (15%) | 14,647 (5.1%) | 6,712 (5.0%) | 3,156 (4.8%) | 3,556 (5.3%) |
| Platelet count < 150 K/uL* | 52,400 (16%) | 51,716 (18%) | 23,887 (18%) | 12,365 (19%) | 11,522 (17%) |
| Temperature < 36.0 or >38.0 C* | 7,818 (2.3%) | 45,974 (14%) | 20,535 (14%) | 10,561 (14%) | 9,974 (13%) |
| Heart rate > 90 beats per minute* | 6,911 (2.1%) | 158,823 (49%) | 74,478 (49%) | 36,848 (49%) | 37,630 (49%) |
| Respiratory rate > 20 breaths per minute* | 8,386 (2.5%) | 81,122 (25%) | 37,505 (25%) | 18,892 (25%) | 18,613 (24%) |
| Systolic blood pressure<90 (mmHg)* | 6,969 (2.1%) | 18,845 (5.8%) | 8,852 (5.8%) | 4,475 (5.9%) | 4,377 (5.7%) |
| Pulse oximetry <90% or on supplemental O2* | 51,459 (15%) | 58,284 (21%) | 27,345 (21%) | 13,691 (21%) | 13,654 (21%) |
| Median (IQR) Body Mass Index* | 14,339 (4.3%) | 28 (24, 33) | 28 (24, 33) | 28 (24, 33) | 28 (24, 33) |
| Admitting location | 0 (0%) |  |  |  |  |
| ICU |  | 52,637 (16%) | 24,836 (16%) | 12,262 (16%) | 12,574 (16%) |
| Observation |  | 65,552 (20%) | 29,588 (19%) | 15,283 (20%) | 14,305 (18%) |
| Ward |  | 215,068 (65%) | 101,172 (65%) | 49,755 (64%) | 51,417 (66%) |
| Outcomes |  |  |  |  |  |
| NV-HAP by electronic surveillance criteria | 0 (0%) | 1,922 (0.6%) | 857 (0.6%) | 430 (0.6%) | 427 (0.5%) |
| NV-HAP by coding/claims definition | 0 (0%) | 2,386 (0.7%) | 1,149 (0.7%) | 584 (0.8%) | 565 (0.7%) |
| 30-day mortality | 0 (0%) | 16,607 (5.0%) | 8,015 (5.2%) | 4,016 (5.2%) | 3,999 (5.1%) |
| Median (IQR) hospital length-of-stay | 0 (0%) | 4 (3, 7) | 4 (3, 7) | 4 (3, 7) | 4 (3, 7) |
| ^1^Median (IQR); n (%) | | | | | |
